# Enhanced noninvasive imaging of oncology models using the NIS reporter gene and bioluminescence imaging

**DOI:** 10.1038/s41417-019-0081-2

**Published:** 2019-01-24

**Authors:** Rianna Vandergaast, Sarawut Khongwichit, Huailei Jiang, Timothy R. DeGrado, Kah-Whye Peng, Duncan R. Smith, Stephen J. Russell, Lukkana Suksanpaisan

**Affiliations:** 1 Imanis Life Sciences, Rochester, MN 55902 USA; 2 Department of Molecular Medicine, Mayo Clinic, Rochester, MN 55905 USA; 3 Institute of Molecular Biosciences, Mahidol University, Salaya, Nakhon Pathom 73170 Thailand; 4 Deparment of Radiology, Mayo Clinic, Rochester, MN 55905 USA

**Keywords:** Cancer imaging, Animal disease models, Cancer models

## Abstract

Noninvasive bioluminescence imaging (BLI) of luciferase-expressing tumor cells has advanced pre-clinical evaluation of cancer therapies. Yet despite its successes, BLI is limited by poor spatial resolution and signal penetration, making it unusable for deep tissue or large animal imaging and preventing precise anatomical localization or signal quantification. To refine pre-clinical BLI methods and circumvent these limitations, we compared and ultimately combined BLI with tomographic, quantitative imaging of the sodium iodide symporter (NIS). To this end, we generated tumor cell lines expressing luciferase, NIS, or both reporters, and established tumor models in mice. BLI provided sensitive early detection of tumors and relatively easy monitoring of disease progression. However, spatial resolution was poor, and as the tumors grew, deep thoracic tumor signals were massked by overwhelming surface signals from superficial tumors. In contrast, NIS-expressing tumors were readily distinguished and precisely localized at all tissue depths by positron emission tomography (PET) or single photon emission computed tomography (SPECT) imaging. Furthermore, radiotracer uptake for each tumor could be quantitated noninvasively. Ultimately, combining BLI and NIS imaging represented a significant enhancement over traditional BLI, providing more information about tumor size and location. This combined imaging approach should facilitate comprehensive evaluation of tumor responses to given therapies.

## Introduction

Early evaluation of cancer therapies relies heavily on rodent oncology models to establish the effects of potential treatments on tumor growth and metastases. In the last several decades, noninvasive imaging of reporter gene-expressing tumor cells has become an important technology for pre-clinical oncology studies, providing researchers with a way to track tumor responses to therapies in real-time, longitudinally, in the same animal [1–3]. These studies have predominately utilized optical reporter genes, including firefly luciferase (Fluc) and a variety of fluorescent proteins (FPs). In particular, bioluminescence imaging (BLI) of Fluc has become popular due to its relative ease-of-use and the high sensitivity that it affords [4, 5]. Nevertheless, BLI suffers from poor spatial resolution and limited signal penetration, which reduce the depth of information that can be acquired using BLI [5]. Here, we sought to enhance and refine tumor imaging by combining BLI with high-resolution, tomographic single photon emission computed tomography (SPECT) or positron emission tomography (PET) imaging using the sodium iodide symporter (NIS) reporter gene.

NIS is a transmembrane protein that mediates uptake of iodide and several other anions into cells. The protein is endogenously expressed in the thyroid—where its activity is critical for normal synthesis of thyroid hormones—and to a lesser degree in the gastric mucosa, salivary glands, and lactating mammary glands [6–10]. Importantly for imaging, NIS mediates the uptake of several clinically approved radiotracers. Therefore, cells engineered to express NIS, either directly or as a result of infection with a NIS-expressing vector, can be tracked noninvasively by SPECT or PET imaging [9–13]. NIS has been used to track or assess regenerative cells [14–19], cellular therapies [20, 21], immune cells [22–24], viral vectors [25], oncolytic viruses [11, 26–30], and tumor cells [31–33] in small and large animal models as well as in humans. Moreover, the versatility of NIS to concentrate multiple readily available SPECT and PET radiotracers facilitates its widespread adoption in pre-clinical imaging.

In this study, we used several oncology models to compare BLI and NIS imaging modalities. Many of our studies used 4T1 cells, which are a highly metastatic model for triple-negative stage IV breast cancer, and are frequently tracked noninvasively using BLI. When implanted into immunocompromised mice or syngeneic Balb/c mice, 4T1 cells form spontaneous metastases at multiple sites including the lungs, liver, lymph nodes, bone, and CNS [34, 35], making them an ideal model for comparing imaging modalities at different tissue depths and anatomical locations. We demonstrate here that combination BLI and NIS imaging significantly increases the precision and accuracy of in vivo tumor characterization, while maintaining sensitive and relatively easy tumor detection and monitoring. This dual reporter imaging approach has the potential to enhance pre-clinical studies of cancer therapies by providing the ultimate clarity on tumor responses, while reducing animal use and study costs.

## Materials and methods

### Generation of reporter gene cell lines

Parental tumor cell lines were purchased from ATCC and maintained at 37^o^C/5% CO_2_ in the appropriate media supplemented with 10% fetal bovine serum (Life Technologies, Carlsbad, CA, USA) and 1 × Pen/Strep. Human lung adenocarcinoma A549 cells (ATCC^®^ CCL-185^TM^) and murine Lewis Lung carcinoma LL/2 cells (ATCC^®^ CRL-1642^TM^) were grown in Dulbecco's modification of Eagle medium (Life Technologies), murine mammary carcinoma 4T1 cells (ATCC^®^ CRL-2539^TM^) were grown in RPMI-1640 (Life Technologies), and human lymphoma Nalm6 cells (ATCC^®^ CRL-3273^TM^) were grown in RPMI-1640 supplemented with an additional 10 mm 4-(2-hydroxyethyl)-1-piperazineethanesulfonic acid (HEPES) (Mediatech, Manassas, VA, USA). All parental cell lines were authenticated by short tandem repeat profiling (IDEXX BioResearch, Columbia, MO, USA). Tumor cell lines stably expressing reporter genes were generated by lentiviral vector transduction. The following lentiviral vectors from Imanis Life Sciences (Rochester, MN, USA) were used: LV-Fluc-P2A-Neo (LV011), LV-Fluc-P2A-Puro (LV012), LV-hNIS-IRES-Neo (LV013), LV-mNIS (LV008), LV-mNIS-PGK-Puro (LV022), and LV-Luc2-P2A-hNIS (LV023). Cells were transduced at a multiplicity of infection of 10 in the presence of 8 μg/ml polybrene. Cells transduced with lentiviral vector containing a neomycin (Neo) or puromycin (Puro) resistance gene were selected using G418 or puromycin, respectively. The A549-hNIS-Neo/Fluc-Puro (CL083) and 4T1-Fluc-Neo/mNIS-Puro (CL066) cell lines were generated by subsequent transduction with two separate lentiviral vectors. The LL/2-mNIS (CL114), 4T1-Fluc-Neo (CL126), 4T1-mNIS (CL117), and Nalm6-Fluc-hNIS (CL143) cell lines underwent clonal selection using ClonaCell TCS (Stemcell Technologies, Vancouver, Canada). All of the generated oncology cell lines tested negative for mycoplasma and are available commercially at Imanis Life Sciences.

### In vitro luciferase assays

A549-hNIS-Neo/Fluc-Puro or parental A549 cells were seeded at densities of 2 through 1 × 10^6^ cells/well in 96-well plates. d-luciferin (Gold Biotechnology, St. Louis, MO, USA) was added to wells at a final concentration of 3 mg/ml and luminescence was immediately measured on an Xenogen IVIS Spectrum (Perkin Elmer, Waltham, MA, USA). The total flux (photons/s/cm^2^/sr) of wells was determined using Living Image software (Perkin Elmer). Values represent the mean (± standard error) total flux from three independent experiments performed in triplicate.

### In vitro NIS ^125^I uptake assay

A549-hNIS-Neo/Fluc-Puro cells were seeded in 6-well plates at densities of 10 through 1 × 10^6^ cells/well. To maintain cell viability, parental A549 cells were added to each well so that the total cell density in each well was constant at 1 × 10^6^ cells/well. Cells were allowed to attach overnight and then subject to an ^125^I uptake assay. Briefly, cells were washed once with 10 mM HEPES in Hank’s Balanced Salt Solution (HEPES/HBSS) and incubated with ^125^I in HEPES/HBSS at 37 ^o^C. After 1 h, cells were washed twice with cold HEPES/HBSS and lysed in 1 M sodium hydroxide. Uptake of ^125^I in the cell lysates was quantitated (in CPM) using an ISO Data-10 gamma counter (GMI Inc., Ramsey, MN 55303). Values represent the mean (± standard error) ^125^I uptake in CPM from three independent experiments performed in triplicate.

### In vitro NIS [^18^F]-TFB uptake assay

Increasing numbers of A549-hNIS-Neo/Fluc-Puro cells were mixed with parental A549 cells in microcentrifuge tubes, such that each tube contained a total of 1 × 10^6^ cells. The cells were incubated at 37 ^o^C for 30 min with 2 μCi [^18^F]-TFB in 1 ml of HEPES/HBSS. The cells were centrifuged and washed once with cold HEPES/HBSS. The final cell pellets were immediately imaged on a small animal Inveon Multiple Modality PET/CT scanner (Siemens Medical Solutions USA, Inc., Malvern, PA, USA). CT was performed at 60 kEv, 500 μA, with 400 ms/projection, 180 projections, and bin 4. The effective pixel size was 94.26 μm. PET was performed at  10 min acquisition, 3DRP reconstruction with Colsher filter, and 0.5 cutoff. Co-registered images were rendered and visualized using the PMOD software (PMOD Technologies, Zurich, Switzerland). Values represent the mean (± standard deviation) [^18^F]-TFB uptake in μCi.

### Establishment of in vivo tumor models

All animal procedures were reviewed and approved by the Mayo Clinic Institutional Animal Care and Use Committee. Balb/c (6-week, female), C57Bl/6 (6-week, female), and Fox SCID beige (6-week, male and female) mice were purchased from Envigo (Indianapolis, IN, USA) or Charles River (Wilmington, MA, USA). Prior to implantation, cells were washed once with cold (4 ^o^C) PBS and resuspended in cold PBS at an appropriate concentration based on the route of implantation. To establish the LL/2, 4T1, and Nalm6 experimental metastases models, mice were implanted with 1 × 10^6^ cells via tail vein injection. To establish subcutaneous tumors, mice ( = 5) were implanted in the right hind flank with 2 x 10^6^ cells. Tumor growth and metastases were monitored by BLI and NIS imaging. Tumor volumes were determined by calipers or CT. For caliper determinations, the length and width of each tumor was measured by caliper, and the tumor volume was calculated using the equation *a*^*2*^×*(b/2)*, where *a* is the shortest dimension. In other cases, tumor area was defined based on CT image using PMOD software.

### Noninvasive BLI

Mice received an intraperitoneal injection (3 mg/mouse) of d-luciferin 10 min before imaging. Bioluminescent signal and grayscale photographic images were acquired using a Xenogen IVIS Spectrum instrument and Living Image software. During image acquisition, mice were maintained under general anesthesia with isoflurane. Bioluminescent signal quantification (photons/s/cm^2^/sr) of regions of interest was carried out using Living Image software. Individual images from different time points were cropped and complied using Adobe Photoshop Elements and Adobe Illustrator (Adobe Inc., San Jose, CA, USA).

### Nuclear imaging

For SPECT imaging, mice were injected with 300 μCi of [^99m^Tc]-pertechnetate via tail vein 1 h prior to image acquisition. Imaging was performed in the Mayo Clinic Small Animal Imaging Core Facility using a U-SPECT-II/CT scanner (MILabs, Utrecht, The Netherlands). Scan volumes for both the SPECT and CT were selected based on orthogonal optical images provided by integrated webcams. Micro-CT image acquisition was performed in 4 min, for normal resolution (169-μm square voxels, 640 slices) at 0.5 mA and 60 kV. Image acquisition time was ~ 20 min for SPECT (69 projections at 50 seconds per bed position). All pinholes focused on a single volume in the center of the tube; by using an XYZ stage, large volumes up to the entire animal were scanned at uniform resolution [36]. Coregistration of the SPECT and CT images was performed by applying pre-calibrated spatial transformation to the SPECT images to match with the CT images. SPECT reconstruction was performed using a POSEM (pixel-based ordered subset expectation maximization) algorithm [37] with six iterations and 16 subsets. CT data were reconstructed using a Feldkamp cone beam algorithm (NRecon v1.6.3, Skyscan). After reconstruction, SPECT images were automatically registered to the CT images according to the pre-calibrated transformation, and re-sampled to the CT voxel size. Co-registered images were further rendered and visualized using the PMOD software. A 3D-Guassian filter (0.8 mm full-width at half maximum) was applied to suppress noise, and LUTs (Look Up Tables) were adjusted for good visual contrast. Reconstructed images were visualized as both orthogonal slices and maximum intensity projections. Maximal intensity projection videos and three-dimensional renderings of regions of interests were performed on the PMOD software.

For PET imaging, mice received 300 μCi of [^18^F]-TFB [38] 45 min prior to image acquisition. PET/CT imaging was performed on a small animal Inveon Multiple Modality PET/CT scanner. CT was performed at 80 kEv, 500 μA, with 250 ms/projection, 180 projections, and bin 4; the effective pixel size was 94.59 μm. PET was performed using 10 min acquisition, OSEM2D reconstruction with Fourier rebinning, and four iterations. Co-registered images were rendered and visualized using the PMOD software.

In order to improve tumor visualization, signals in the thyroid, salivary glands, and stomach owing to endogenous NIS, and in the bladder owing to secreted radiotracer were removed from images using PMOD software.

## Results

### NIS radiotracer uptake correlates with cell number

**Fig. 1:**
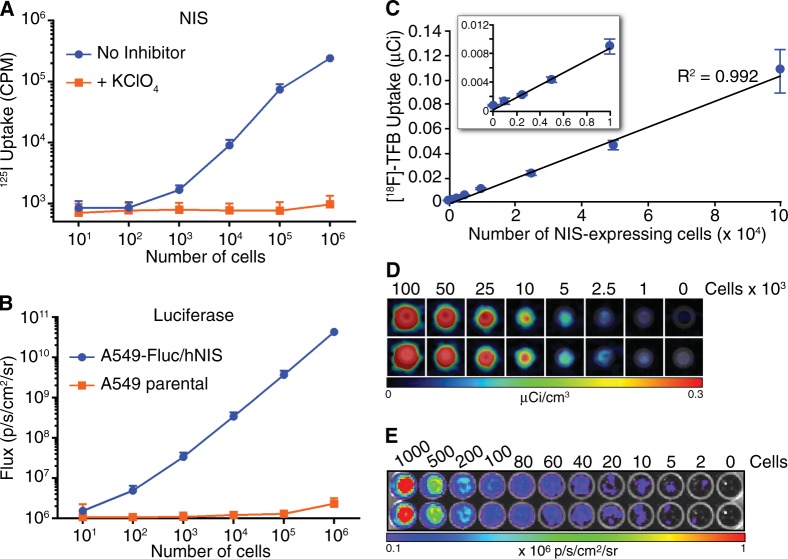
In vitro reporter activity of A549-hNIS-Neo/Fluc-Puro cells. **a** The indicated number of A549-hNIS-Neo/Fluc-Puro cells were seeded in six-well plates and an ^125^I uptake assay was performed in the presence or absence of the NIS inhibitor KClO_4_. **b** The indicated number of A549-hNIS-Neo/Fluc-Puro or parental A549 cells were seeded in 96-well plates, and luminescence (Flux) was measured immediately after the addition of 3 mg/ml d-luciferin. **c**, **d** The indicated number of A549-hNIS-Neo/Fluc-Puro cells were mixed with parental A549 cells to a total of 1 × 10^6^ cells/tube and incubated for 30 min with [^18^F]-TFB. Following a wash to remove residual [^18^F]-TFB, the cell pellets were imaged by PET/CT **d** and the [^18^F]-TFB uptake from each tube was determined **c**. **e** The indicated number of A549-hNIS-Neo/Fluc-Puro cells were seeded in 96-well plates, and BLI was performed immediately after the addition of 3 mg/ml d-luciferin

The main objective of this study was to define a method for improved accuracy and precision in pre-clinical tumor imaging by using the NIS reporter to enhance standard BLI. To this end, we first generated tumor cell lines expressing NIS and firefly luciferase (Fluc), and characterized NIS and luciferase signal in vitro. NIS activity correlated strongly with cell number in vitro, except at very low numbers of NIS-expressing cells (Fig. 1a). Fluc activity also correlated strongly to cell number over a wide cell range (Fig. 1b). To determine the in vitro sensitivity of NIS imaging by PET, uptake of radiotracer [^18^F]-TFB in cell pellets of NIS-expressing cells was imaged using PET. For these experiments, each cell pellet contained a total of 1 × 10^6^ cells, but the number of NIS-expressing cells was increased from 1 × 10^3^ to 1 × 10^6^ cells. The cells were incubated with [^18^F]-TFB for 30 min, before being washed and pelleted for imaging. [^18^F]-TFB uptake directly correlated with cell number from 1 × 10^3^ to 1 × 10^6^ cells (Fig. 1c and data not shown). Although we detected 1000 NIS-expressing cells in one tube, 2500 NIS-expressing cells was required for consistent detection (Fig. 1d), indicating the sensitivity of PET imaging was approximately 2500 NIS-expressing cells in a cell pellet of 1 × 10^6^ cells. By comparison, as few as 2 Fluc-expressing cells could be consistently detected using BLI (Fig. 1e). Taken together, these findings demonstrated that reporter signal intensity is an accurate indicator of cell number for both NIS and luciferase over a wide cell range. Moreover, both BLI and NIS imaging offer sensitive detection of reporter cells. Although luciferase offers superior sensitivity in vitro, it is subject to tissue attenuation, which can rapidly reduce sensitivity in vivo at increased tissue depths.

### High-resolution NIS imaging facilitates precise tumor localization for discrimination of deep-tissue tumors

**Fig. 2:**
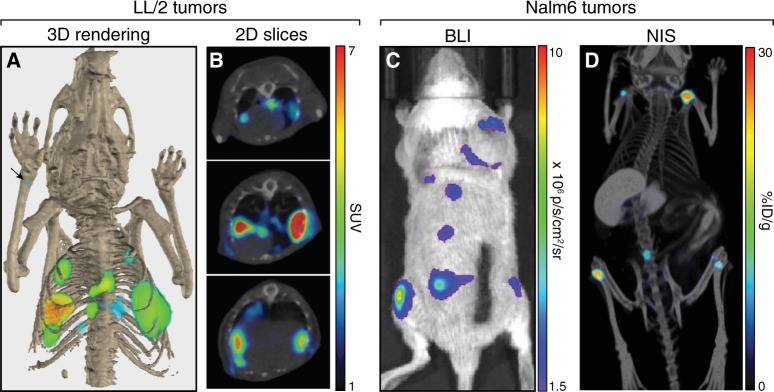
High-resolution tumor imaging with precise anatomical localization using NIS. **a**, **b** C57Bl/6 mice were implanted intravenously with LL/2-mNIS cells; a representative mouse is shown. LL/2-mNIS tumors were imaged by PET/CT using [^18^F]-TFB on day 27. Shown is a 3D rendering of tumor localization within the mouse (arrow indicates location of bone metastasis) **a** and 2D z-stack slices of the thoracic region (3-mm interval) **b**. **c**, **d** SCID beige mice were implanted intravenously with Nalm6-Fluc-hNIS cells; a representative mouse is shown. Nalm6-Fluc-hNIS tumors were imaged by BLI on day 26 **c** and by SPECT/CT using ^125^I on day 25 **d**

Although BLI is frequently used to noninvasively image tumor models, precise imaging and localization of tumor metastases is difficult using BLI due to poor spatial resolution and tissue attenuation of signal that results in surface-weighted images. In contrast, NIS imaging by small animal SPECT or PET produces tomographic images with spatial resolution below 1-mm, dependent on the device used for image acquisition [39, 40]. As a proof of concept, we implanted mice with murine Lewis lung carcinoma LL/2 cells expressing NIS (LL/2-mNIS) and acquired high-resolution tomographic images of the tumors within the mice (Fig. 2a, b). Precise anatomical localization of NIS signal was possible through the combination of PET imaging with X-ray computed tomography (CT). Tumors were primarily observed within the thoracic cavity, though one small bone metastasis was also detected (Fig. 2a; arrow). Precise anatomical localization of NIS reporter signal was also clearly demonstrated in mice implanted with acute lymphoblastic leukemia Nalm6 cells expressing luciferase and NIS (Nalm6-Fluc-hNIS). BLI of these mice (Fig. 2c) revealed a tumor growth pattern consistent with the expected spinal and bone metastases [41–44]. Using SPECT/CT imaging of NIS, we precisely and definitively localized the tumors to the long bones noninvasively (Fig. 2d and S1). NIS imaging, therefore, provided an important level of accuracy and precision for tumor imaging that could not be obtained with BLI alone.

**Fig. 3:**
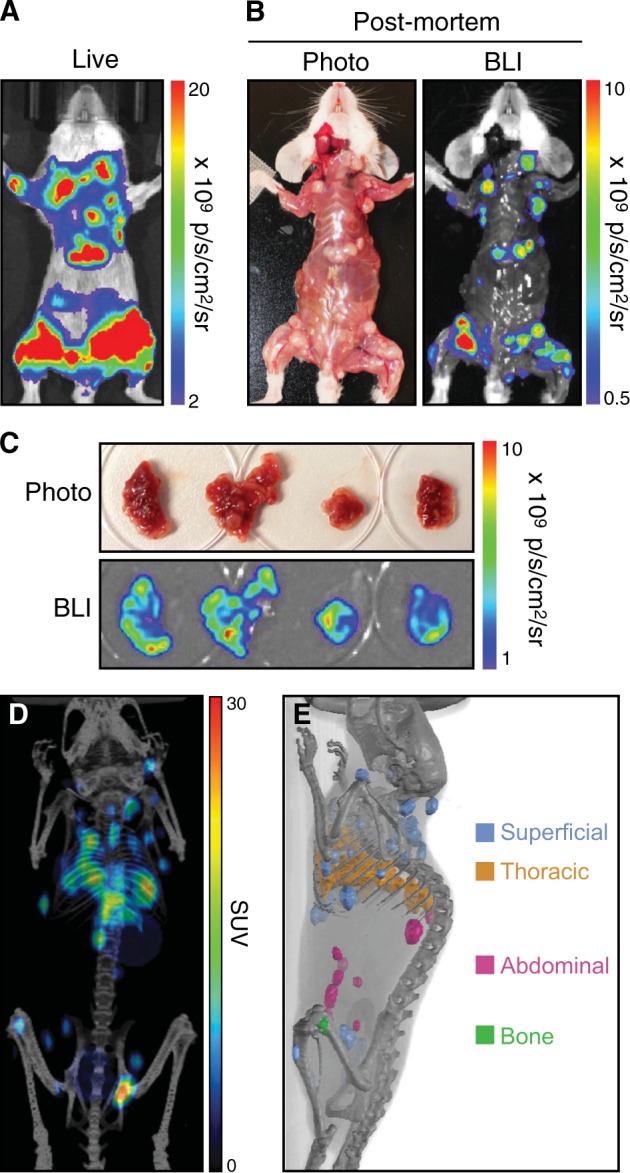
Discrimination of surface and deep-tissue tumors using NIS. **a**–**c** Balb/c mice were implanted intravenously with 4T1-Fluc-Neo cells; a representative mouse is shown. **a** Tumors were imaged by BLI. **b** Post-mortem autopsy and BLI was performed upon killing and removal of the skin. **c** Excised lung lobes were imaged by BLI. **d**, **e** Balb/c mice were implanted intravenously with 4T1-mNIS cells and PET/CT imaging was performed after 20 days using [^18^F]-TFB; a representative mouse is shown. **d** Global [^18^F]-TFB uptake in mice. **e** Tomographic representation of tumors from **d**, colored based on tumor locations

The importance of high spatial resolution combined with precise anatomical localization for tumor imaging was further demonstrated when we imaged mice with mammary carcinoma 4T1 tumors. A significant global tumor burden was detected in mice implanted with luciferase-expressing 4T1-Fluc cells (Fig. 3a), though precise localization was not possible. Moreover, superficial luciferase signals from subcutaneous tumors masked the deep-tissue thoracic signals. Little or no luciferase signal was observed in the lungs of killed mice (Fig. 3b), though numerous tumors were readily detected by BLI when the lungs were excised (Fig. 3c). In contrast, in mice implanted with NIS-expressing 4T1-mNIS cells, thoracic, abdominal, bone, and superficial subcutaneous 4T1-mNIS tumors were readily distinguished by PET/CT imaging of NIS (Fig. 3d, e and S2). Thus, compared to BLI, NIS imaging provided additional clarity of tumor burden, tumor location, and individual tumor distribution within the animals.

### Noninvasive quantitation of NIS signal correlates strongly to tumor volume

**Fig. 4:**
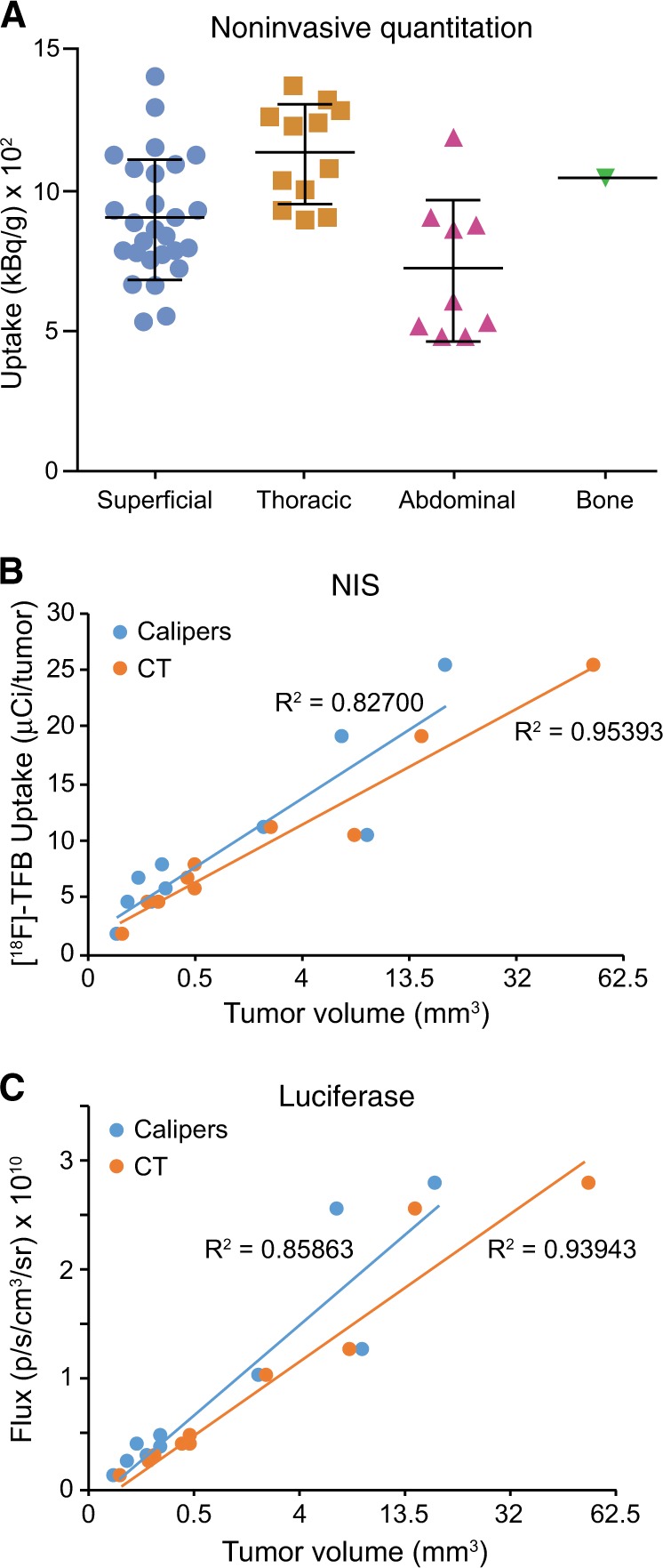
In vivo correlation of Fluc and NIS signal to tumor volume. **a** Median uptake (kBq/g) of [^18^F]-TFB from individual 4T1-mNIS tumors from mouse in Fig. 3d and e. **b**, **c** Five SCID beige mice were implanted subcutaneously with 4T1-Fluc-Neo/mNIS-Puro cells. On days 9 and 16 after implantation tumors were measured with calipers and BLI imaging was performed. SPECT/CT imaging using [^99m^Tc]-pertechnetate was performed on days 10 and 17 after implantation. Tumor volumes determined by caliper measurements or CT were compared to [^99m^Tc]-pertechnetate uptake **b** and total tumor flux **c**

In pre-clinical imaging studies, reporter signal is frequently used to quantitate tumor burden. However, luciferase and other optical reporters are subject to tissue attenuation, which makes accurate quantitation difficult for non-surface tumors. As radiotracer signal is not attenuated by tissues, we were able to noninvasively quantitate NIS signal in the tumor-bearing mice at all tissue depths. Furthermore, the high-resolution images obtained using NIS imaging facilitated quantification of individual tumor nodules (Fig. 4a). [^18^F]-TFB uptake was similar in all tumor types, though thoracic tumors exhibited slightly higher radiotracer uptake, whereas abdominal tumors exhibited slightly lower radiotracer uptake. Whether these differences have biological significance is unknown.

To determine whether NIS radiotracer uptake accurately predicts tumor volume, we measured NIS reporter signal from subcutaneous tumors. Mice were implanted with 4T1-Fluc-Neo/mNIS-Puro cells, which express both luciferase and NIS, in the right hind flank. SPECT/CT imaging was used to measure NIS signal ([^99m^Tc]-pertechnetate uptake) from each tumor, whereas calipers and CT were used to calculate tumor volumes. [^99m^Tc]-pertechnetate uptake over the tumor correlated closely with tumor volume (Fig. 4b), demonstrating consistency of NIS-dependent radiotracer uptake over a large range of tumor volumes. We also examined the relationship of luciferase signal to tumor volume using BLI. Fluc activity (total flux) correlated closely with tumor volume (Fig. 4c), consistent with previous reports that luciferase signal accurately reflects tumor volume for surface tumors [19, 45, 46].

### Combination BLI and NIS imaging offers high sensitivity, resolution, and precise anatomical localization

**Fig. 5:**
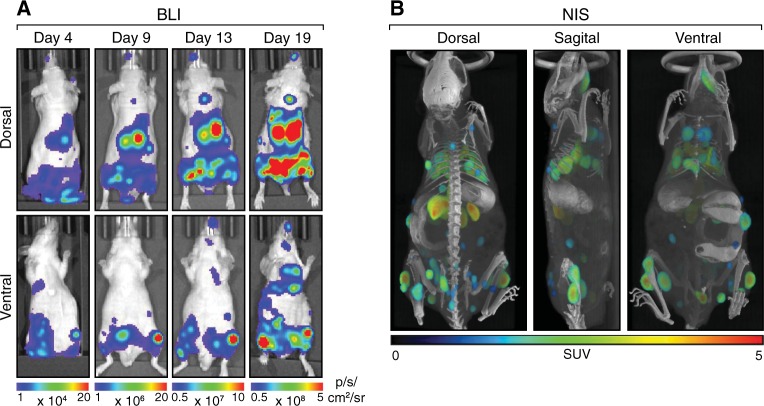
Multi-modality BLI and NIS imaging of tumors. SCID beige mice were implanted intravenously with 4T1-Fluc-Neo/mNIS-Puro cells; a representative mouse is shown. **a** Tumors were tracked over time using BLI. **b** On day 20 after implantation, PET/CT imaging was performed using [^18^F]-TFB

Our results demonstrated that NIS imaging of tumors provides several advantages over BLI, including high spatial resolution of individual tumor nodules, precise anatomical localization of tumors, and the capacity to more accurately detect and noninvasively quantitate deep-tissue tumors. Nonetheless, BLI offers several benefits over NIS imaging, including its high sensitivity, relative ease-of-use, relative speed, and the lower cost of imaging. Therefore, a multi-modality combination of BLI and NIS imaging should represent an enhancement over tumor imaging using either reporter alone. To evaluate combined BLI and NIS imaging, we implanted mice intravenously with 4T1-Fluc-Neo/mNIS-Puro cells and performed BLI and NIS imaging of the resulting tumors. Using BLI we readily detected tumors early after implantation (Day 4; earliest time examined) (Fig. 5a). Moreover, three mice were imaged (dorsal and ventral) within 10 min, making this a convenient way to quickly asses and monitor tumor burden at earlier time points. When tumor burden became prominent, NIS imaging by PET/CT using [^18^F]-TFB afforded high spatial resolution for the discrimination of individual tumor nodules (Fig. 5b and S3), which was not possible by BLI. NIS imaging also facilitated precise anatomical localization and quantification of the tumors. Thus, multi-modality BLI and NIS imaging offers both high sensitivity and resolution, and maintains much of the ease-of-use of BLI while accurately detecting and quantitating deep-tissue tumors.

## Discussion

Noninvasive tracking of tumors through BLI has played an important role in pre-clinical evaluation and development of cancer therapies. Here, we demonstrated that BLI of tumors can be enhanced by using multi-modality imaging with NIS. As a reporter gene, NIS offers several advantages relative to BLI. NIS imaging by small animal SPECT or PET produces tomographic images with spatial resolution below 1-mm (range 0.9 mm to 0.16 mm), dependent on the scanner and collimator used for image acquisitions [39, 40]. Moreover, when used in combination with CT, precise anatomical localization of signal is possible. NIS-expressing tumors can be readily detected at most anatomical locations, though background NIS signal can limit detection of tumors in the thyroid, salivary glands, mammary glands, and stomach. As radiotracer signal is not subject to tissue attenuation in mice, or can be corrected for attenuation, imaging and noninvasive quantitation of deep-tissue tumors is possible. Compared with other nuclear reporters, NIS also offers more versatility as a reporter gene due to the ready availability of a number of radiotracers for NIS imaging. Thus, NIS imaging is well-suited to complement standard BLI. Indeed, several groups have demonstrated the benefits of combining BLI with imaging of nuclear reporter genes (NIS, SSTR2, HSVTK) for tracking immune cells, regenerative cells, or therapeutic cells in pre-clinical studies [16, 19, 22, 47–51].

Many key questions in pre-clinical oncology research center around tumor growth and metastasis, and the effect of potential therapies on these processes. Noninvasive imaging allows researchers to answer these questions more effectively and accurately in living animals. Tumor growth and the formation of metastases can be tracked in the same animal over time. This tracking facilitates more accurate disease modeling, better equipping researchers to determine when, if at all, to begin treatment studies. Moreover, therapy responses can be evaluated in real-time, and residual disease can be easily detected and tracked in the animals. Given the widespread application of noninvasive imaging in the field of oncology, the advancement of methods, such as that described here, for more accurately imaging tumors is highly desirable.

Although BLI offers superior sensitivity to NIS for imaging of superficial tissues, use of the recently developed PET tracer [^18^F]-TFB offers increased sensitivity and quantitative accuracy for imaging NIS in deep tissues [38, 52, 53]. Using [^18^F]-TFB we detected as few as 1000 NIS-positive cells (Fig. 1d). Likewise, Fruhwirth and colleagues were able to detect 2000 NIS-positive cells within a volume of 5 × 10^5^ cells [31]. In our studies, NIS imaging also provided superior sensitivity to CT imaging alone, which was unable to detect most soft tissue tumors in the tumor-bearing mice. Recently, NIS was used to track the fate of therapeutic CAR-T cells in mice [21]. Moreover, because NIS is directly clinically translatable and has therapeutic potential itself [54–58], the use of NIS for tracking therapeutic cells both pre-clinically and clinically is likely to rise in the future. For this application, advances in the sensitivity of NIS imaging will provide a clear advantage. Although advances may occur in the form of further radiotracer development or more sensitive equipment, modulation of NIS itself may also contribute to increased sensitivity. Because sensitivity is directly related to NIS or luciferase expression and activity within the cells, sensitivity can be improved by increasing protein expression, or increasing enzyme or transporter activity.

Just as advances have been made in NIS imaging, new technologies have improved signal quantitation and localization by BLI. Newer optical imagers (e.g., the Perkin Elmer IVIS^®^ SpectrumCT, and Bruker InVivo X-treme II), combine BLI with CT or X-ray imaging to improve anatomical localization of signal. The development of more advanced computer algorithms has also increased the accuracy of BLI signal quantification by accounting more for signal depths. Despite these advances, however, signal attenuation still prevents precise anatomical localization and accurate quantitation of deep-tissue tumors by BLI alone.

Combining BLI with NIS imaging exploits the strengths of both imaging technologies (Table 1). BLI can be used for sensitive and relatively easy monitoring of tumor formation and growth. NIS imaging at key intervals provides tomographic, high-resolution images that are fully quantitative, and anatomically localized. Moreover, by combining modalities in a single animal, this superior clarity can be achieved using fewer animals, saving time and money while accelerating therapy evaluation.

**Table 1:** Comparison of the strengths and weaknesses of BLI and NIS Imaging

Parameter	BLI	NIS Imaging
Equipment	Optical Imager	SPECT or PET
Spatial Resolution	Several mm	Less than 1 mm (dependent on equipment)
Sensitivity	Highest (less than 10 cells in vitro) (depth dependent in vivo)	High (1000-2000 cells in vitro) (depth independent in vivo)
Depth of signal	~1 cm (exponential signal attenuation with depth)	No limit (minimal signal attenuation with depth)
Signal quantitation	Surface-weighted	Accurate at all tissue depths
Throughput	High (~1-2 min per scan up to 5 mice simultaneously)	Low (~15-20 min per scan 1-4 mice simultaneously)

## Supplementary information

Supplemental legends

Supplemental video S1

Supplemental video S2

Supplemental video S3
