# Supplementary material for: Enhanced noninvasive imaging of oncology models using the NIS reporter gene and bioluminescence imaging
Source: Cancer Gene Ther. 2019 Jan 24;27(3):179–88. doi: 10.1038/s41417-019-0081-2 (PMC7170803; doi:10.1038/s41417-019-0081-2)
Supplement: Supplementary file 1 — Supplemental legends [file 41417_2019_81_MOESM1_ESM.docx]

**SUPPLEMENTAL MOVIES**

**Supplemental S1**: SCID beige mice were implanted intravenously with Nalm6-Fluc-hNIS cells. Nalm6-Fluc-hNIS tumors were imaged by SPECT/CT using ^125^I on day 25. A 3D tomographic movie of a representative mouse is shown (same mouse as in Figure 2D).

**Supplemental S2**: SCID beige mice were implanted intravenously with 4T1-mNIS cells. 4T1-mNIS tumors were imaged by SPECT/CT using [^99m^Tc]-pertechnetate on day 14. A 3D tomographic movie of the thoracic region of a representative mouse is shown; in addition to tumor signal (blue/green), lungs are shown shaded in grey, the heart and blood vessels are shown in red, and the skeleton is shown in beige.

**Supplemental S3:** SCID beige mice were implanted intravenously with 4T1-Fluc-Neo/mNIS-Puro cells. On day 20 after implantation, PET/CT imaging was performed using [^18^F]-TFB. A 3D tomographic movie of a representative mouse is shown (same mouse as in Figure 5B).
